# The density and spatial tissue distribution of CD8^+^ and CD163^+^ immune cells predict response and outcome in melanoma patients receiving MAPK inhibitors

**DOI:** 10.1186/s40425-019-0797-4

**Published:** 2019-11-15

**Authors:** Daniela Massi, Eliana Rulli, Mara Cossa, Barbara Valeri, Monica Rodolfo, Barbara Merelli, Francesco De Logu, Romina Nassini, Michele Del Vecchio, Lorenza Di Guardo, Roberta De Penni, Michele Guida, Vanna Chiarion Sileni, Anna Maria Di Giacomo, Marco Tucci, Marcella Occelli, Francesca Portelli, Viviana Vallacchi, Francesca Consoli, Pietro Quaglino, Paola Queirolo, Gianna Baroni, Fabrizio Carnevale-Schianca, Laura Cattaneo, Alessandro Minisini, Giuseppe Palmieri, Licia Rivoltini, Mario Mandalà, on behalf of the Italian Melanoma Intergroup, Sara Simi, Fabio Galli

**Affiliations:** 1 Section of Pathological Anatomy, Department of Health Sciences, University of Florence, Florence, Italy; 2 Department of Oncology, Istituto di Ricerche Farmacologiche Mario Negri IRCCS, Milan, Italy; 3 Unit of Immunotherapy of Human Tumors, Fondazione IRCCS Istituto Nazionale dei Tumori, Milan, Italy; 4 Department of Pathology, Fondazione IRCCS Istituto Nazionale dei Tumori, Milan, Italy; 5 Unit of Medical Oncology, Department of Oncology and Hematology, Papa Giovanni XXIII Hospital, Bergamo, Italy; 6 Unit of Clinical Pharmacology and Oncology, Department of Health Sciences, University of Florence, Florence, Italy; 7 Unit of Oncology, Fondazione IRCCS Istituto Nazionale dei Tumori, Milan, Italy; 8 Department of Oncology, Hematology, and Respiratory Diseases, University Hospital of Modena, Modena, Italy; 9 Department of Medical Oncology and Molecular Genetics Laboratory, IRCCS Istituto Tumori Giovanni Paolo II, Bari, Italy; 10 Melanoma and Esophageal Cancer Unit, Istituto Oncologico Veneto-IRCCS, Department of Medical Oncology, Padua, Italy; 11 Medical Oncology and Immunotherapy, Center for Immuno-Oncology, University Hospital of Siena, Istituto Toscano Tumori, Siena, Italy; 12 Medical Oncology Unit, Department of Biomedical Sciences and Human Oncology, University of Bari ‘Aldo Moro’, Bari, Italy; 13 Azienda Ospedaliera Santa Croce e Carle di Cuneo SC Oncologia, Cuneo, Italy; 14 Department of Oncology, ASST Spedali Civili, Brescia, Italy; 15 Department of Medical Sciences, Section of Dermatology, University of Turin, Turin, Italy; 16 Unit of Medical Oncology, Ospedale Policlinico San Martino, Genoa, Italy; 17 Medical Oncology Candiolo Cancer Institute-FPO, IRCCS, Candiolo, Italy; 18 Division of Pathological Anatomy, Papa Giovanni XXIII Hospital, Bergamo, Italy; 19 Department of Oncology, Azienda Sanitaria Universitaria Integrata di Udine, Udine, Italy; 20 Unit of Cancer Genetics, Institute of Biomolecular Chemistry, National Research Council, Sassari, Italy

**Keywords:** Myeloid cells, T lymphocytes, Microenvironment, Melanoma prognosis

## Abstract

**Background:**

Clinical response to MAPK inhibitors in metastatic melanoma patients is heterogeneous for reasons still needing to be elucidated. As the patient immune activity contributes to treatment clinical benefit, the pre-existing level of immunity at tumor site may provide biomarkers of disease outcome to therapy. Here we investigated whether assessing the density and spatial tissue distribution of key immune cells in the tumor microenvironment could identify patients predisposed to respond to MAPK inhibitors.

**Methods:**

Pretreatment tumor biopsies from a total of 213 patients (158 for the training set and 55 for the validation set) treated with BRAF or BRAF/MEK inhibitors within the Italian Melanoma Intergroup were stained with selected immune markers (CD8, CD163, β-catenin, PD-L1, PD-L2). Results, obtained by blinded immunohistochemical scoring and digital image analysis, were correlated with clinical response and outcome by multivariate logistic models on response to treatment and clinical outcome, adjusted for American Joint Committee on Cancer stage, performance status, lactate dehydrogenase and treatment received.

**Results:**

Patients with high intratumoral, but not peritumoral, CD8^+^ T cells and concomitantly low CD163^+^ myeloid cells displayed higher probability of response (OR 9.91, 95% CI 2.23–44.0, *p* = 0.003) and longer overall survival (HR 0.34, 95% CI 0.16–0.72, *p* = 0.005) compared to those with intratumoral low CD8^+^ T cells and high CD163^+^ myeloid cells. The latter phenotype was instead associated with a shorter progression free survival (*p* = 0.010). In contrast, PD-L1 and PD-L2 did not correlate with clinical outcome while tumor β-catenin overexpression showed association with lower probability of response (OR 0.48, 95% CI 0.21–1.06, *p* = 0.068).

**Conclusions:**

Analysis of the spatially constrained distribution of CD8^+^ and CD163^+^ cells, representative of the opposite circuits of antitumor vs protumor immunity, respectively, may assist in identifying melanoma patients with improved response and better outcome upon treatment with MAPK inhibitors. These data underline the role of endogenous immune microenvironment in predisposing metastatic melanoma patients to benefit from therapies targeting driver-oncogenic pathways.

## Introduction

Approximately 40–50% of metastatic melanoma patients (MPs) harbor point mutations in *BRAF*, over 95% of which are at V600 in *BRAF* exon 15 [1]. The discovery of this mutation provided the genetic basis for the development of BRAF inhibitors (BRAFi) for the treatment of melanoma. Clinical efficacy of this class of drugs was initially demonstrated by their use in mono-therapy in patients with *BRAF* ^V600^-mutant melanoma. In two prospective randomized clinical trials BRAFi showed a better response rate, progression free survival (PFS) and overall survival (OS) than chemotherapy [2, 3]. However, responses were temporally limited, mainly because of acquired resistance. Improvement of efficacy and tolerability was attained with dual MAPK pathway inhibition by adding a MEK inhibitor (MEKi) to a BRAFi as reported in phase 3 randomized studies [4–6]. Therefore, BRAFi/MEKi combination has been recommended as a standard therapy for advanced *BRAF* ^V600^-mutated melanoma, being associated with a median PFS and OS of 12 months and 24–36 months, respectively [4–6]. Albeit the problem of overcoming primary and acquired resistance still needs to be faced for therapeutic amelioration, about 30–35% of patients are alive at 5 years indicating the onset of long-term tumor control [7]. The identification of biomarkers that predict durable benefit in patients with BRAF^V600^-mutated melanoma would provide essential tools for better treatment personalization.

Beside the effect on the biological target and pathway, there is strong evidence that the therapeutic efficacy of BRAFi and MEKi relies on additional factors involved in tumor-host interactions and preclinical data show that oncogenic BRAF contributes to immune evasion, as targeting this mutation may increase melanoma immunogenicity [8].

Several genomic mechanisms of intrinsic or acquired tumor resistance to MAPKi therapies have been reported, including BRAF^V600^ amplification and single nucleotide variants in NRAS, KRAS, MEK1/2, PTEN, CDKN2A and DUSP4 [9]. A study comparing the genomic features of complete responders (CR) versus fast progressors (PD) in patients treated with BRAFi/MEKi showed higher rates of MITF amplification and TP53 mutation in PD, whereas NF1 deletion and deleterious mutations were more common in CR [10]. Nevertheless, gene signatures of CD8 T effector cells, cytolytic T-cells, antigen presentation and NK cells were significantly enriched in CR tumors [10]. Indeed, several evidences support a key role of tumor immunity in the therapeutic efficacy of MAPKi. LEF1 down-expression and β-catenin induction, which reduce T cells and CD103^+^ dendritic cells tumor infiltrate via inhibition of CCL4 secretion [11], have been reported to promote acquired resistance to BRAFi and MEKi [12]. A rapid accrual of activated CD8^+^ T cells in tumor microenvironment is instead triggered by BRAFi administration at early time points [13], in association with clinical benefit [14]. Preclinical studies linked this effect to the upregulation of HLA molecule expression in tumor cells, favoring increased antigen presentation and activation of antitumor T cells, together with the downregulation of certain immunosuppressive factors such as PD-L1, IL1, IL8, NT5E, and VEGFA [15]. On the other hand, non responding patients are featured by the accrual in the tumor site and peripheral blood of myeloid immunosuppressive cell elements and macrophages [16], again pointing to immunity as a key player to MAPKi therapeutic activity.

Based on these data, we designed a study aimed at identifying essential tissue immune biomarkers able to capture the immune contexture of tumor microenvironment that could potentiate or contrast the clinical efficacy of MAPKi.

## Materials and methods

### Patient characteristics

The cohort of the training set (*n* = 158) was identified by inspecting the electronic databases of all metastatic MPs treated at Italian Melanoma Intergroup (IMI) centers from June 2011 to February 2017. We retrieved data concerning clinical outcome and MAPKi treatment from patients enrolled in compassionate, expanded access use protocols or therapeutic use of BRAFi with or without MEKi since 2011. The local Ethics Committees approved the study protocol. The study was conducted in compliance with the World Medical Association Declaration of Helsinki. Patients enrolled in the study were treated with vemurafenib or vemurafenib and cobimetinib within the therapeutic and expanded access use according to clinical practice. For patients included in the vemurafenib compassionate program use, the inclusion criteria were an Eastern Cooperative Oncology Group performance status (ECOG-PS) 0–2, as well as normal hepatic (serum bilirubin < 1.5 mg/dl), renal (serum creatinine < 1.5 mg/dl) and bone marrow (leucocyte count > 4000/1 l, platelet count > 100,000/1 l) functions. For the other patients, exclusion criteria were a rapid deteriorating medical condition, with severe liver or renal failure, QTc > 500 mS and ECOG-PS 4. Information on age, gender, histopathology, and surgical and medical treatment were retrieved for each patient, as well as data on tumor objective response rate (ORR), PFS and OS. Data on treatment and survival were collected prospectively. Medical records and/or review of pathology material confirmed accuracy in histopathological classification. Tumor stage was assessed according to the American Joint Committee on Cancer (AJCC) TNM (Tumor, Node, Metastasis) staging system classification (VII edition). Clinical response to BRAFi/MEKi was assessed by RECIST v1.1 criteria [17].

Patients of the validation set (*n* = 55) were instead treated at the Istituto Nazionale dei Tumori of Milan, with BRAFi according to the MO25515 trial (multicenter phase II study on first−/second-line Vemurafenib; ClinicalTrials.gov, NCT01307397) [18] (*n* = 35) or BRAFi/MEKi by clinical practice (*n* = 20). Similarly to the training cohort ECOG-PS was 0–2, normal hepatic (serum bilirubin < 1.5 mg/dl), renal (serum creatinine < 1.5 mg/dl) and bone marrow (leucocyte count > 4000/1 l, platelet count > 100,000/1 l) functions was required to receive targeted therapy. Information on demographics was retrieved for each patient, as well as data on PFS and OS. Data on treatment and survival were collected prospectively.

### Tissue samples

Formalin fixed paraffin-embedded (FFPE) tissue sections, 4 μm in thickness, were stained with hematoxylin and eosin and centrally reviewed to confirm the histopathological diagnosis and to assess pathology tissue quality control.

### Immunohistochemistry

Representative 4-μm thick FFPE tissue sections of pre-treatment melanoma samples were selected for immunohistochemical analysis. Sections were incubated with the following primary antibodies: CD8 (rabbit monoclonal CONFIRM, clone SP57 ready to use; Ventana Medical Systems, Tucson, AZ), CD163 (mouse monoclonal, clone 10D6, dilution 1:100, Novocastra Laboratories Ltd., Newcastle, UK), β-catenin (mouse monoclonal, clone 14 ready to use, Ventana Medical Systems, Tucson, AZ), PD-L1 (rabbit monoclonal, clone E1L3N, dilution 1:50, Cell Signaling, Danvers, USA) and PD-L2 (rabbit monoclonal, clone D7U8C, dilution 1:50, Cell Signaling, Danvers, USA) on a Ventana BenchMark ULTRA immunostainer (Ventana Medical Systems, Tucson, AZ). The staining procedure included pretreatment with cell conditioner 1 followed by incubation with the different antibodies. For all antibodies, the signal was developed with the Universal Red Detection Kit (Ventana Medical Systems, Tucson, AZ). Sections were then counterstained with hematoxylin. Tissue sections of tonsil were used as positive control. As negative controls, mouse IgG1 isotype control was used for β-catenin and CD163 while rabbit IgG isotype control was used for CD8, PD-L1 and PD-L2, respectively. The control sections were treated in parallel with the samples.

Immunohistochemical scoring was performed in a blinded fashion by experienced melanoma pathologists (DM, MC, BV). Stained sections were initially assessed at low magnification to select the areas with highest density of positive immune cells at peritumoral and intratumoral location. Assessment of CD8^+^ T lymphocytes and CD163^+^ macrophages score density was compared with evaluation obtained by image analysis. Evaluation of tumoral β-catenin and PD-L1 was performed as previously described [19, 20]. PD-L2 expression was evaluated on tumor cells. Assessment of the training set was centralized in the University of Florence, while the validation set was evaluated at the Istituto Nazionale dei Tumori of Milan, according to shared standard operating procedures.

### Digital image acquisition and analysis

Tissue sections stained with CD8 and CD163 antibodies were digitally scanned at an absolute magnification of X200 using D-Sight platform (A. Menarini Diagnostic, Florence, Italy). An algorithm was designed based on pattern recognition that quantified CD8^+^ and CD163^+^ cells within two tumor compartments: the invasive tumor margin (stromal-tumor edge) and inside the tumor parenchyma (tumor center). Image analysis based on RGB (red, green, blue) spectra was used to detect all cells by counterstaining with hematoxylin (blue) and fast red. The number of fast red CD8^+^ and CD163^+^ cells was calculated in five different high-power magnification fields of 10^− 3^ mm^2^. The algorithm calculated the density of CD8^+^ and CD163^+^ cells/mm^2^. The total number of CD8^+^ and CD163^+^cells was then calculated as the mean of each high-power magnification field. CD8 and CD163 expression was determined using two read-outs that were independent of each other to account for tumor heterogeneity.

**Fig. 1:**
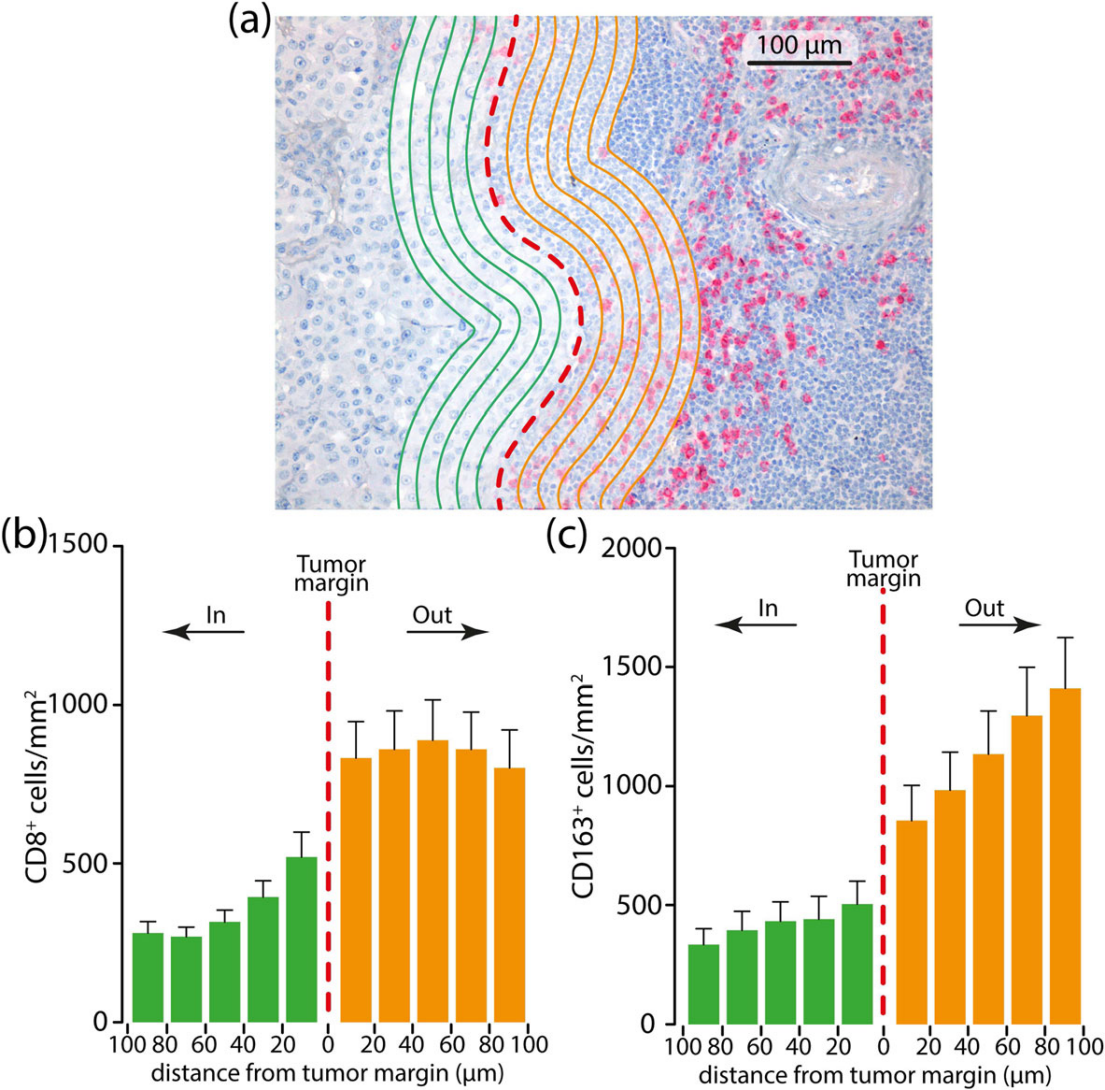
Representative metastatic melanoma tissue with analysis mark-up (**a**). Panel A illustrates a CD8 stain; red dashed line is the invasive tumor margin. CD8^+^ and CD163^+^ cells are counted within the invasive margin, 100 μm inside and 100 μm outside the tumor as identified with green and orange lines spaced 20 μm. **b**, **c** CD8^+^ and CD163^+^ cells’ density is binned according to the distance from the margin and a 20 μm bin histogram is generated. The middle of the histogram if the tumor boundary (red dashed line), to the left is inside tumor (green bars) and to the right is outside tumor (orange bars)

The immune cell density (CD8^+^ and CD163^+^ cells) at the peritumoral area was further explored in order to generate a cell density histogram. The peritumoral compartment was defined as the region centered on the border separating the host tissue from the malignant nests, with an extent of 500 μm. To analyze further the spatial distribution of CD8^+^ and CD163^+^ cells in the peritumoral area, an algorithm was designed to create thick bands (1 mm^2^) 20 μm internal and external to the tumor margin. Then, the distribution of the CD8^+^ and CD163^+^ cells related to the tumor margin was identified in consecutive 20 μm steps (distance classes) within 100 μm (Fig. 1).

### Statistical analysis

PFS was defined as the time from the date of start treatment to the date of progression or death from any cause whichever comes first. Patients who did not progress or die at the date of analysis were censored at their last disease assessment date. OS was defined as the time from the date of start treatment to the date of death from any cause. Overall response rate (ORR) was defined as the proportion of patients with a complete or partial response to treatment. Survival curves were estimated with the Kaplan-Meier method. PFS and OS were analyzed by means of Cox regression model and results were expressed as hazard ratios (HR) with their 95% confidence intervals (95% CI). ORR was analyzed by means of logistic regression models and expressed as odds ratios (OR) with their 95% CI. All multivariate models included as covariates immunohistochemical variables, AJCC stage, performance status, lactate dehydrogenase (LDH) and the treatment received (BRAFi+MEKi vs BRAFi alone). OS multivariable models included also the subsequent treatment (immunotherapy vs no immunotherapy).

PD-L1 and PD-L2 were tested as a continuous or as a dichotomous variable using 5% as cut-off. Score density of CD8^+^ T cells and CD163^+^ macrophages in intratumoral and peritumoral location was assessed as follows: 0, absent; 1+, mild (< 10%); 2+, moderate (10–50%); 3+, marked (50–100%) and their density was evaluated as dichotomous variable as high (2+, 3+) versus low (0, 1+). β-catenin was tested as a continuous or as a dichotomous variable using the median value as cut-off. CD8^+^ T cells and CD163^+^ macrophages were also analyzed in combination, categorizing patients in three groups: group 1, high CD8^+^ T cells and low CD163^+^ macrophages; group 2, high CD8^+^ T cells and high CD163^+^ macrophages/low CD8^+^ T cells and low CD163^+^ macrophages; group 3, low CD8^+^ T cells and high CD163^+^ macrophages. CD8^+^ T cells and immunohistochemical PD-L1 overexpression were combined in three groups as follow: group 1, PD-L1 ≥ 5% and low CD8^+^ T cells; group 2, PD-L1 ≥ 5% and high CD8^+^ T cells/PD-L1 < 5% and low CD8^+^ T cells; group 3, PD-L1 < 5% and high CD8^+^ T cells. Combining β-catenin expression and CD8^+^ T cells, patients were categorized in three groups: group 1, low CD8^+^ T cells and β-catenin overexpressed; group 2, high CD8^+^ T cells and β-catenin overexpressed/low CD8^+^ T cells and β-catenin not overexpressed; group 3, high CD8^+^ T cells and β-catenin not overexpressed.

Chi square test was used to assess associations between PD-L1, PD-L2, β-catenin, CD8^+^ and CD163^+^ status and other clinical and pathological features. The Kruskal-Wallis test was used to analyze the association between the cell count and the density score in CD8^+^ T cells and CD163^+^ macrophages.

To test the robustness of the results, an independent series of metastatic MPs was analyzed separately as validation cohort. The validation cohort included metastatic MPs who received BRAFi or BRAFi plus MEKi at the Istituto Nazionale dei Tumori of Milan; their inclusion and exclusion criteria were the same as those for the training set.

All tests were two sided and the statistical significance was set at < 0.05 for each analysis. Statistical analyses were carried out using SAS version 9.4 (SAS Institute, Cary, NC) and R language environment for statistical computing (open source, www.r-project.org version 3.4.3).

## Results

### Patients and treatments

Demographic and clinical characteristics of the training set included are summarized in Additional file 1: Table S1. 158 patients were enrolled in the training set; 60 % of patients were male, and the median age at diagnosis of metastatic disease was 59 years (Q1-Q3: 47.7–70.7). All patients had metastatic disease, 60% with M1c disease (95 patients). One hundred and thirty-six patients (86%) and 22 patients (14%) received MAPKi as 1st- or 2nd-line therapy, respectively. Ninety-four patients (60%) received BRAFi as a monotherapy, while 64 patients (40%) received BRAFi+MEKi. The most frequent subsequent lines of treatment were immunotherapy and chemotherapy in 25 and 17% of patients, respectively. Approximately 56% of patients were not treated with further treatments because of rapid progressive disease.

Patients of the validation set were comparable to the training set cohort for demographics and clinical parameters. Thirty patients (55%) were male; all patients had metastatic disease and 55% with M1c disease (30 patients). Thirty-five patients (64%) received BRAFi as a monotherapy, while 20 patients (36%) received BRAFi+MEKi. Twelve patients (22%) received immunotherapy as a subsequent line of therapy.

### Immunohistochemical β-catenin, PD-L1, PD-L2, CD8 and CD163 expression in melanoma samples

**Fig. 2:**
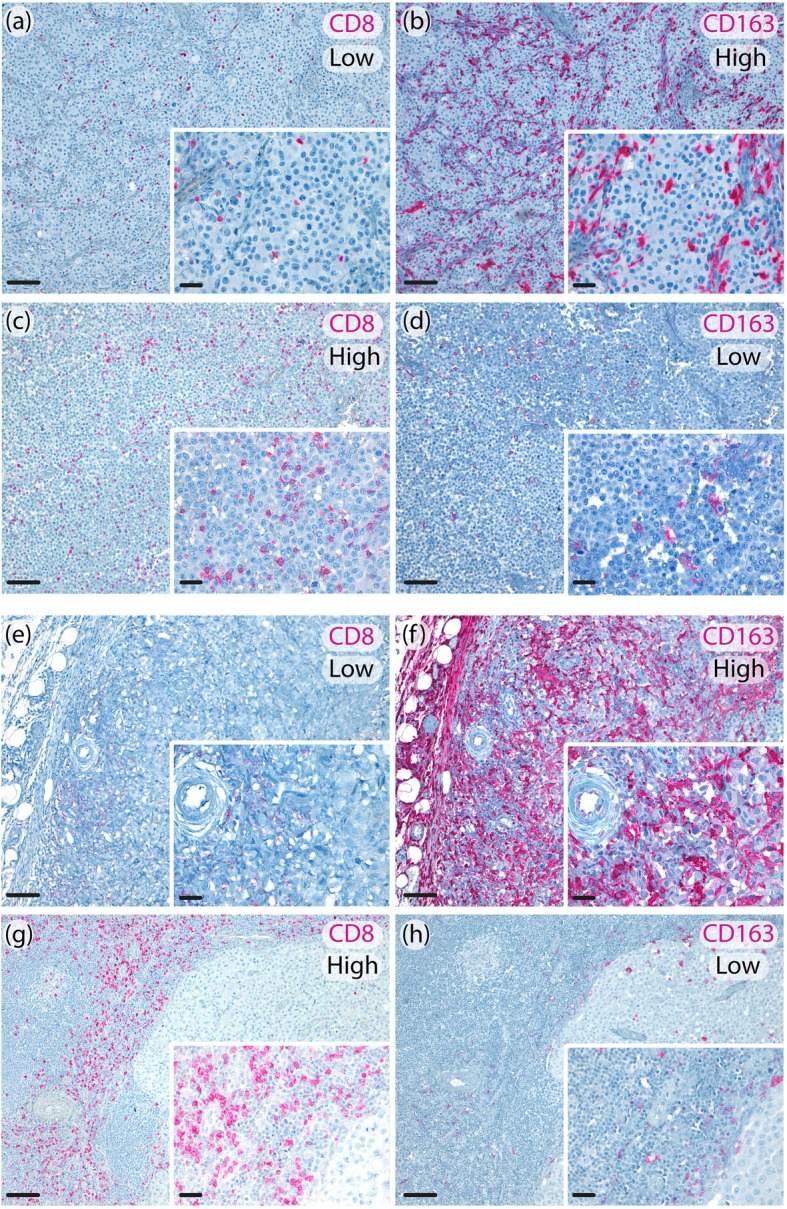
Representative metastatic melanoma tissues showing intratumoral low CD8^+^/ high CD163^+^ expression (**a**, **b**); intratumoral high CD8^+^/low CD163^+^ expression (**c**, **d**). (original magnification 10x, scale bar 100 μm, insert 40x, scale bar 20 μm); peritumoral low CD8^+^/high CD163^+^ expression (**e**, **f**); peritumoral high CD8^+^/low CD163^+^ expression (**g**, **h**). (original magnification 10x, scale bar 100 μm, insert 40x, scale bar 20 μm)

A panel of representative immune markers was tested by immunohistochemistry on melanoma biopsies from the training set, including PD-L1 and PD-L2 (included as surrogates of inflammed tumors and tumor immune escape), β-catenin (chosen as tumor pathway driving immune-suppressed microenvironment), CD8 (as marker of antitumor effector T cells) and CD163 (recapitulating tumor associated myeloid cells including macrophages). Immune markers expression was evaluated in the last available metastatic sample before starting MAPKi therapy in 122 patients (Fig. 2, Additional file 2: Fig. S1 and S2). In the remaining cases, biomarkers were evaluated in the primary melanoma samples due to unavailability of metastatic tissue. The median interval between metastatic biopsies and treatment starting was 3 months (range 1–6 months). PD-L1 expression on the tumor cell membrane was negative in 82 patients (57%), positive in 63 patients (43%) and technically not evaluable (NE) in 15 patients, while PD-L2 was negative in 126 patients (89%), positive in 15 (11%) patients and NE in 18 patients. The median expression of β-catenin was 60% (interquartile range (IQR): 20–80, NE, in 9 patients), 0 (IQR: 0–0, N NE A: 11 patients) and 10 (IQR: 0–80, NE: 9 patients) for membranous, nuclear and cytoplasmic expression, respectively. These values were used as cut-offs to analyze β-catenin as dichotomous variable.

The expression of PD-L1 was associated with high intratumoral CD163^+^ macrophages (*p* = 0.008) and high peritumoral CD163^+^ cells (*p* = 0.032), conversely PD-L1 expression was not associated neither with intratumoral nor with peritumoral CD8^+^ T-cell melanomas (Additional file 1: Table S2).

Density and spatial distribution of the above mentioned immune markers were then divided in discrete categories, and their prevalence is reported in Additional file 1: Table S3 and Additional file 2: Figure S3.

### The impact of tissue biomarkers on ORR

**Fig. 3:**
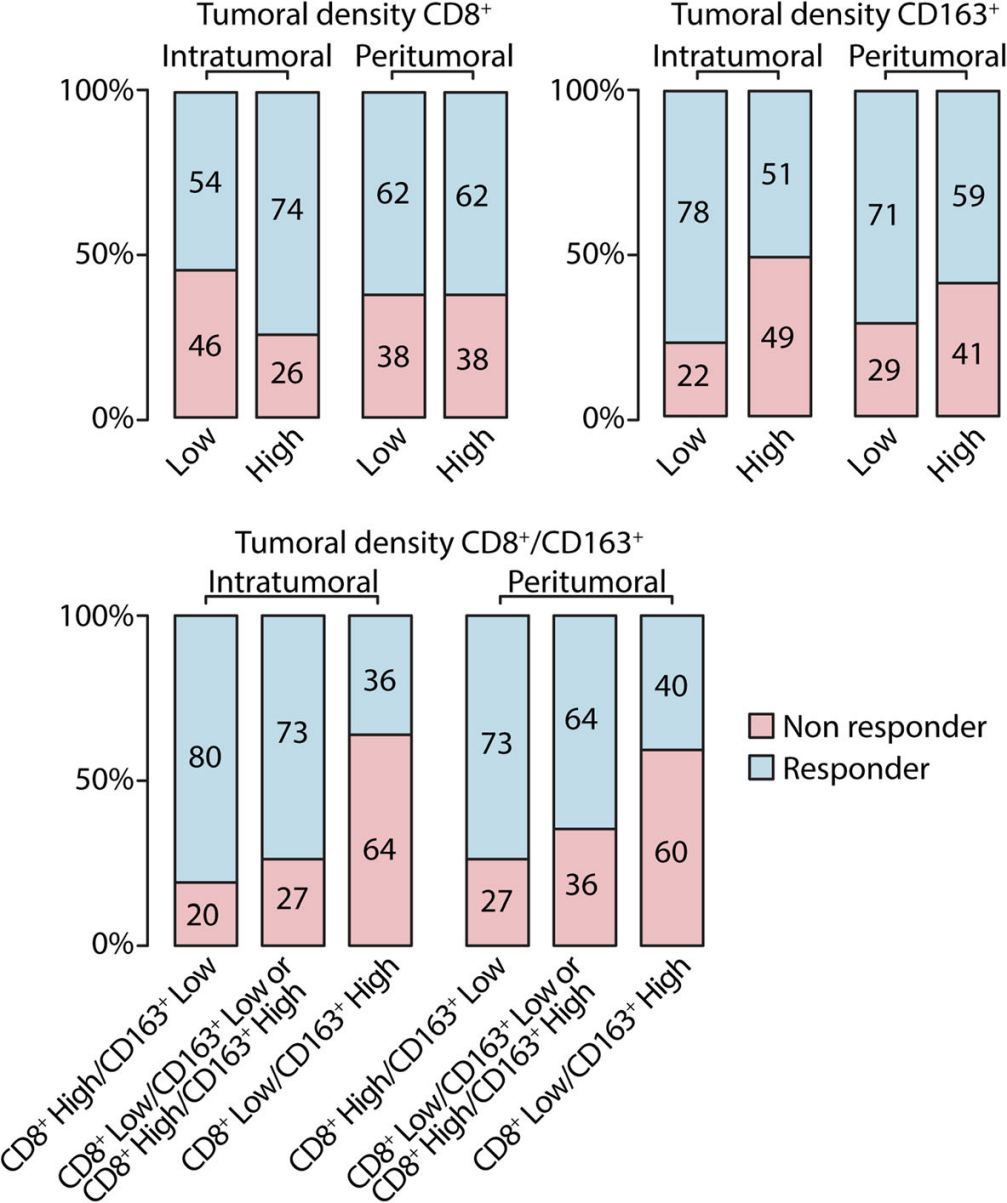
Response to treatment according to intratumoral and peritumoral density CD8^+^ T cells and CD163^+^ macrophages. Low: score = 0, 1+; High: score = 2+,3+; non responder: patients who have experienced a stable or progressive disease as best response to therapy; responder: patients who have experienced a complete or partial response as best response to therapy

Treatment response was available for 156 patients and included 26 (16.7%) complete responses; 73 (46.8%) partial responses; 25 (16.0%) stable disease and 32 (20.5%) progressive disease. The distribution of responder patients according to intra and peritumoral CD8^+^ T-cell and CD163^+^ macrophages density is reported in Fig. 3.

Results of multivariate logistic models on response to treatment, adjusted for AJCC stage, performance status, LDH and treatment received (BRAFi+MEKi vs BRAFi), are reported in Additional file 1: Table S4. Metastatic MPs with high intratumoral CD8^+^ T-cell count (OR 2.15 95% CI 0.93–4.98, *p* = 0.074) had a higher probability of response to treatment, while those with membranous β-catenin overexpression > 60% (OR 0.48, 95% CI 0.21–1.06, *p* = 0.068) showed a lower probability of response. Metastatic MPs with high intratumoral CD163^+^ count (OR 0.28, 95% CI 0.12–0.65, *p* = 0.003) had a statistically significant lower probability of response, while the same profile (high CD163^+^ macrophages) in the peritumoral space did not reach any statistical difference (*p* = 0.136) (Additional file 1: Table S4). The rate of CR was 24% vs 4% among patients with high CD8^+^/low CD163^+^ immunophenotype, respectively (*p* = 0.04).

Furthermore, a statistically significant higher probability of response was observed in patients with β-catenin negative and high intratumoral CD8^+^ T-cell count compared to those with β-catenin overexpression and low intratumoral CD8^+^ melanomas (Additional file 1: Table S4).

Interestingly, when patients were analyzed according to the combined evaluation of the intratumoral and peritumoral density of CD8^+^ and CD163^+^ cells, a higher probability of response was observed in patients with high intratumoral, but not peritumoral, CD8^+^ T cells and low CD163^+^ macrophages compared to those with low intratumoral CD8^+^ T cells and high intratumoral CD163^+^ macrophages (OR 9.91, 95% CI 2.23–44.0, *p* = 0.003) (Additional file 1: Table S4).

### The impact of tissue biomarkers on PFS and OS

At a median follow-up of 34 months, 121 (78.1%) patients had progressed and 109 (69.0%) had died. Overall, 126 (79.7%) patients progressed or died. The median PFS and OS were 8.3 (IQR: 4.6–19.2) and 13.7 (IQR: 6.1–38.6) months, respectively.

**Fig. 4:**
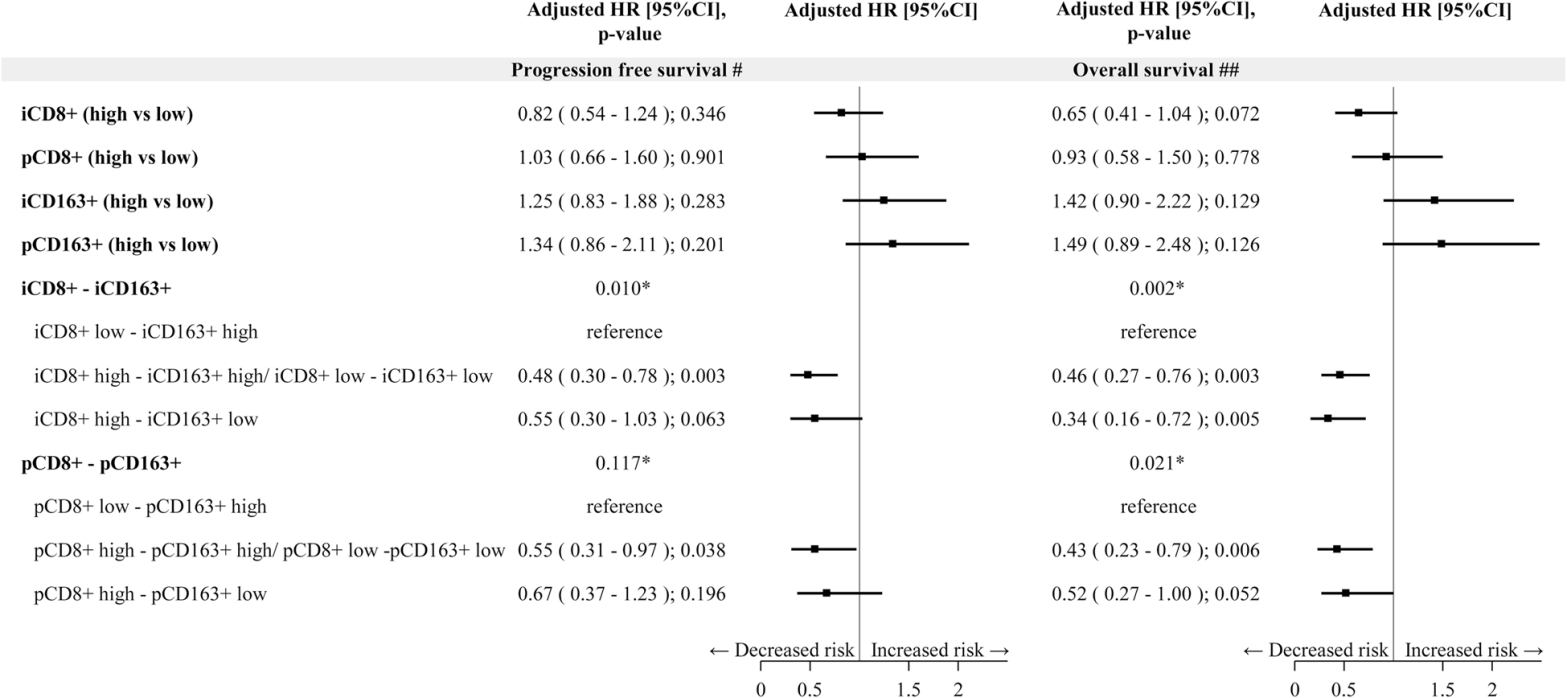
Forest plot on progression free survival and overall survival -Multivariable Cox regression model – Impact of tissue biomarkers on progression free survival and overall survival. Legend: ^#^Adjusted for Stage, LDH, PS, treatment (BRAFi+MEKi vs BRAFi); ^##^ Adjusted for Stage, LDH, PS, treatment (BRAFi+MEKi vs BRAFi), subsequent Immunotherapy (yes/no); i: intratumoral; p: peritumoral; CD8^+^/ CD163^+^ low: score 0,1+, high: score 2+,3 +

**Fig. 5:**
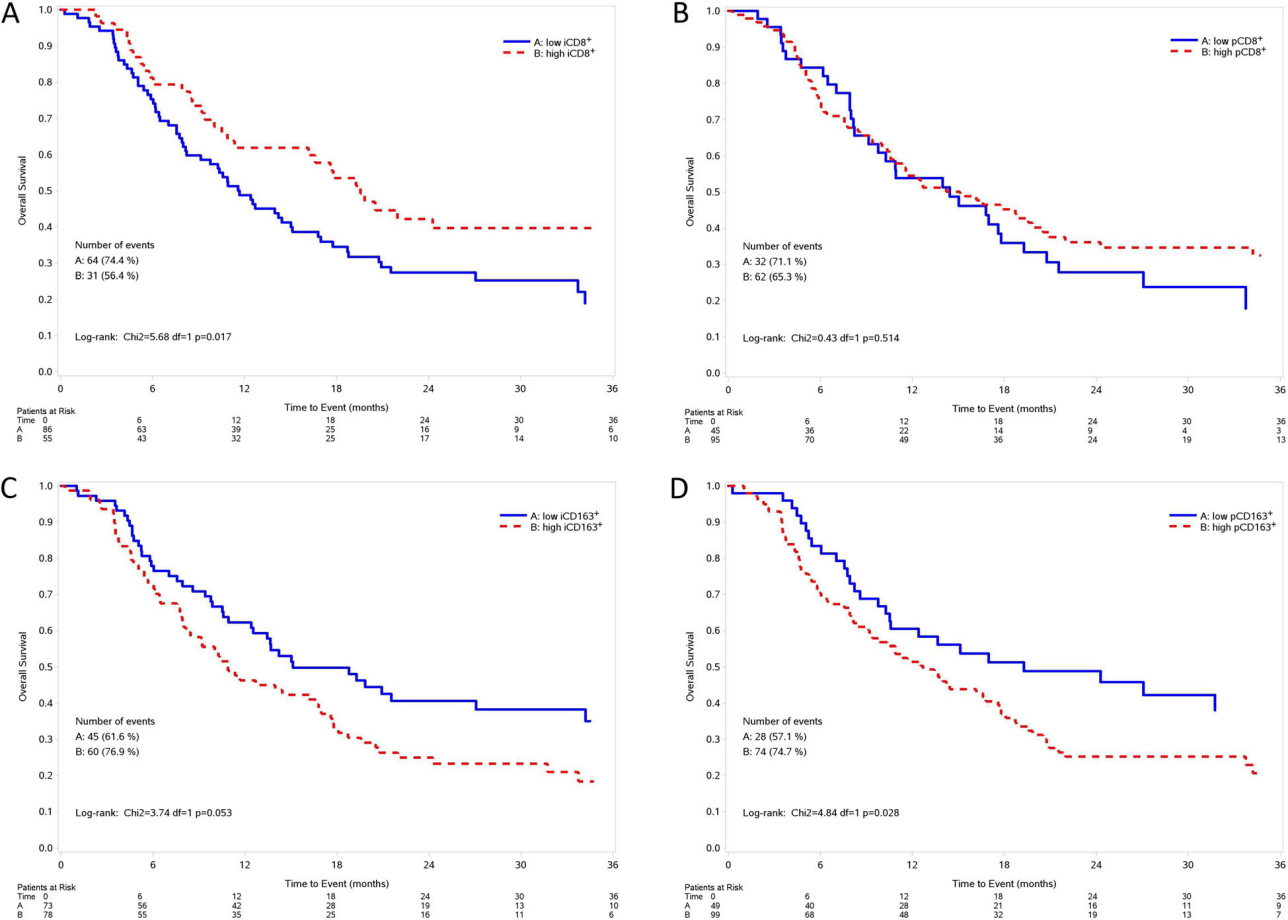
Kaplan-Meier curves for overall survival according to intratumoral CD8^+^ T cells (**a**), peritumoral CD8^+^ T cells (**b**), intratumoral CD163^+^macrophages (**c**), peritumoral CD163^+^ macrophages (**d**). Low: score = 0, 1+; High: score = 2+,3+; iCD8^+^: intratumoral CD8^+^; pCD8^+^: peritumoral CD8^+^; iCD163^+^: intratumoral CD163^+^; pCD163^+^: peritumoral CD163^+^

**Fig. 6:**
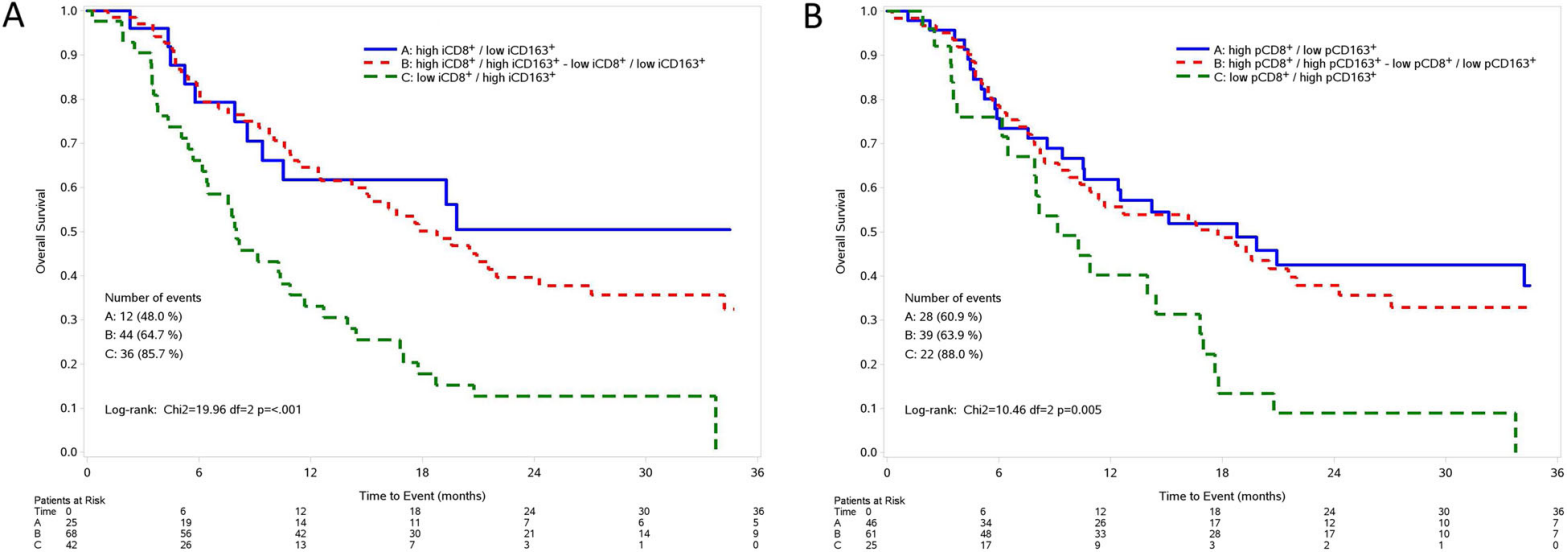
Kaplan-Meier curves for overall survival according to the combination of intratumoral (**a**) and peritumoral (**b**) CD8^+^ T cells and CD163^+^ macrophages. Low: score = 0, 1+; High: score = 2+,3+; iCD8^+^: intratumoral CD8^+^; pCD8^+^: peritumoral CD8^+^; iCD163^+^: intratumoral CD163^+^; pCD163^+^: peritumoral CD163^+^

Results of multivariate analysis, both for PFS and OS are reported in Fig. 4 and Additional file 1: Table S5. At multivariate assessment, a shorter PFS was observed in patients with intratumoral, but not peritumoral, low CD8^+^ T cells and high CD163^+^ macrophages (*p* = 0.010) (Fig. 4, Additional file 1: Table S5). At multivariate analysis, after adjusting for stage, LDH, PS, treatment received (BRAFi+MEKi vs BRAFi), subsequent immunotherapy (yes/no), metastatic MPs with high intratumoral, but not peritumoral, CD8^+^ T cells density showed a barely detectable statistically significant difference in terms of OS (HR 0.65, 95% CI 0.41–1.04, *p* = 0.072) (Fig. 4). Notably, patients with high intratumoral, but not peritumoral, CD8^+^ T cells and low intratumoral CD163^+^ macrophages (HR 0.34, 95% CI 0.16–0.72, *p* = 0.005) had a longer OS compared to those with intratumoral low CD8^+^ T cells and high CD163^+^ macrophages. Figure 5 and Fig. 6 show Kaplan-Meier curves for OS according to CD8^+^ T cell and CD163^+^ macrophages alone or in combination, respectively.

### Validation cohort: impact of tissue biomarkers on PFS and OS

Patients of the validation case set (*n* = 55) were enrolled and evaluated independently at the Istituto Nazionale dei Tumori of Milan. Patient demographic and clinical characteristics are summarized in Additional file 1: Table S6.

At a median follow-up of 41.5 months, 45 (81.8%) patients had progressed and 12 (21.8%) had died. Overall, 45 (78.2%) patients progressed or died. The median PFS was 9.3 (IQR: 5.8–48.0), while the median OS was not reached.

**Fig. 7:**
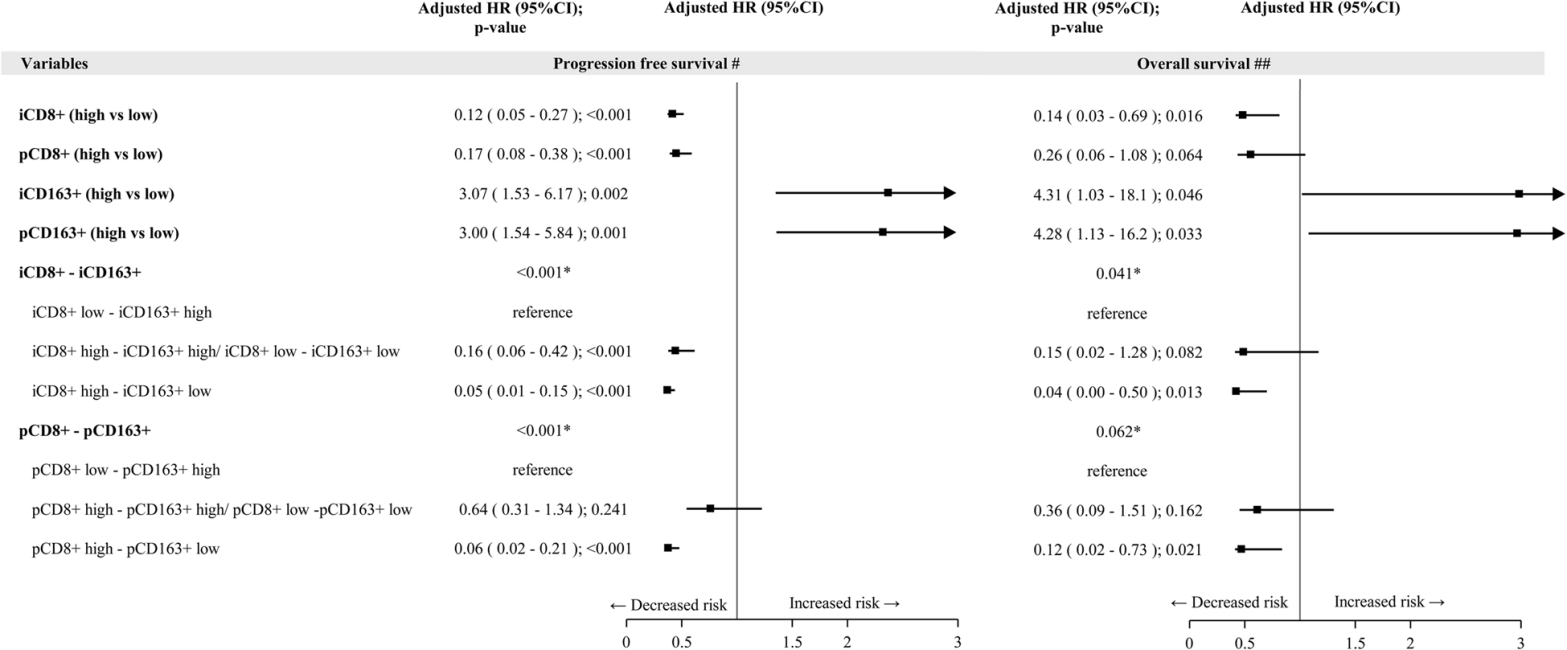
Forest plot on progression free survival and overall survival in the validation cohort. Multivariable Cox regression model – Impact of tissue biomarkers on progression free survival and overall survival.^#^Adjusted for Stage, treatment (BRAFi+MEKi vs BRAFi); ^##^ Adjusted for Stage, treatment (BRAFi+MEKi vs BRAFi), subsequent Immunotherapy (yes/no); i: intratumoral; p: peritumoral; CD8^+^/ CD163^+^ low: score 0,1+, high: score 2+,3 +

Results of multivariate analysis for PFS and OS are reported in Fig. 7. At multivariate assessment, after adjusting for stage, treatment received (BRAFi+MEKi vs BRAFi), a shorter PFS was observed in patients with intratumoral low CD8^+^ T cells and high CD163^+^ macrophages (*p* < 0.001 and *p* = 0.002 for CD8^+^ and CD163^+^, respectively) (Fig. 7). Regarding OS, at multivariate analysis, after adjusting for stage, treatment received (BRAFi+MEKi vs BRAFi) and subsequent immunotherapy (yes vs no), metastatic MPs with high intratumoral, but not peritumoral, CD8^+^ T cells density showed a statistically significant better prognosis (HR 0.14, 95% CI 0.03–0.69, *p* = 0.016 for intratumoral and HR 0.26, 95% CI 0.06–1.08, *p* = 0.064 for peritumoral CD8^+^ T cells) (Fig. 7). Notably, patients with high intratumoral CD8^+^ T cells and low intratumoral CD163^+^ macrophages (HR 0.04, 95% CI 0.00–0.50, *p* = 0.013) had a longer OS compared to those with intratumoral low CD8^+^ T cells and high CD163^+^ macrophages (Fig. 7).

## Discussion

Increasing evidence indicates that response and long-term outcome to treatment with MAPKi in melanoma patients is influenced by clinical prognostic parameters related mostly to tumor burden and aggressiveness features. While the initial clinical response to MAPKi primarily relies on the loss of kinase activity of ERK, subsequent adaptive events appear to be mediated by the intervening action of immune cells. Accordingly, strategies to improve long-term responses to MAPKi necessarily require a better understanding of the diverse cellular patterns of the complex tissue microenvironment (TME). In this timely context of clinical and translational research, the three most striking findings of this study are: 1) metastatic MPs with absent/low infiltration of CD8^+^ T cells and a high density of CD163^+^ macrophages at intratumoral, but not peritumoral location, had a statistically significant shorter OS compared to those with high density CD8^+^ T cells and absent/low density of CD163^+^ macrophages. 2) metastatic MPs with absent/low intratumoral CD8^+^ T cells and high intratumoral CD163^+^ macrophages showed a nearly statistically significant shorter PFS compared to those with the opposite profile, while the same profile (low CD8^+^ T cells/high CD163^+^ macrophages) in the peritumoral space did not exhibit any tendency; 3) the response rate of patients with high intratumor CD163^+^ macrophages was lower than those with absent or low CD163^+^ intratumor infiltration, while the response rate was not affected by changes in the peritumoral CD163^+^ macrophages. Thus, both the density and distribution of CD163^+^ macrophages seem to determine the biological and clinical events associated with ORR. One of the major issues in exploiting MAPKi for metastatic melanoma patients lies in the interpatient degree and duration of response: some patients progress upon treatment, while others achieve complete response, and the remainder is somewhere in between. Hence, there is a clinical need to identify biomarkers that can allow accurate identification of the best treatment approach in the individual patient with BRAF-mutated melanoma. Identifying biomarkers correlated with a higher probability of response and a longer PFS could be clinically and translationally relevant for two main reasons: i) in symptomatic patients or in those who are candidate to a neoadjuvant approach the probability and the degree of response could be important to identify patients who can draw a remarkable and sustained response to treatment, which in turns correlates with a good prognosis; ii) several studies showed that the high and prolonged response correlates with better outcome. The rate of CR is indeed a surrogate biomarker strongly correlated with long term outcome in several prospective studies investigating the efficacy of targeted therapy in melanoma [21, 22].

For this reason we assessed the rate of patients who achieved a complete response to targeted therapy according to the investigated biomarkers in the TME. In our series, the rate of CR was significantly increased in MPs with high CD8^+^/low CD163^+^ versus those with low CD8^+^/high CD163^+^ immunophenotype. Our study, by identifying simple and reliable biomarkers correlated with response and longer PFS, could be translationally and clinically relevant. Reproducible biomarker measurements are essential, particularly for long-term projects with valuable patient samples.

Our results showed an uneven spatial distribution of immune cells in the intra- and peritumoral space, and allowed to combine these cellular biomarkers in biosignatures with opposing roles, favoring or disfavoring response and better prognosis of metastatic MPs treated with BRAFi/MEKi, [13, 14, 23]. We suggest that none of the biomarkers taken individually is able to predict the long-term outcome of patients receiving MAPKi. Only the combination of multiple markers is therefore able to reflect the complexity of the TME and to predict the outcome of patients. Furthermore, our findings support the hypothesis that a more hostile TME at baseline is associated with a worse ORR and outcome in BRAF^V600^-mutated metastatic MPs receiving MAPKi. However, in our cohort, tumor overexpression of PD-L1 or β-catenin in association with intratumoral or peritumoral CD8^+^ T lymphocytes or CD163^+^ was not an independent prognostic factor at multivariate analysis. Consistently with our previous study, we found that a statistically significant higher probability of response was observed in metastatic MPs with β-catenin negative and high intratumoral CD8^+^ T-cell count compared to those with β-catenin overexpression and low intratumoral CD8^+^ melanomas [19]. Nevertheless, we previously reported a better OS in metastatic MPs with high density of CD8^+^ T lymphocytes and no overexpression of β-catenin, than those with no CD8^+^ T lymphocytes and overexpression of β-catenin [19]. Incorporating the evaluation of both CD8^+^ T cells and CD163^+^ macrophages attenuates the predictive power of β-catenin in identifying MAPKi-treated metastatic MPs with better outcome. The key role of CD8^+^ T cells recruited into the tumor compartment is underlined by the adoptive T cell transfer protocols developed in melanoma that have consistently yielded high and durable clinical response in selected patients [24]. However, our data further support the implication of CD163^+^ cells in dominant inhibitory pathways in melanoma, implying that the presence of protumor and immunosuppressive myeloid cells as shutting down their function in TME ultimately favors tumor outgrowth. Our original contribution definitively includes macrophages in this scenario, where conflicting data have been reported so far [25].

The observation in human tumor biopsies from 10 patients treated with vemurafenib or a combination of dabrafenib and trametinib that treatments increased macrophages [26, 27], suggests that macrophages are recruited to the tumor site by BRAFi/MEKi treatment, and that targeting macrophages in combination with BRAFi/MEKi may affect patient response. Tumor-promoting M2 macrophages can contribute to tolerance to MAPK inhibition, and their accumulation within tumors during treatment strongly correlates with an aggressive phenotype in different melanoma models, through different mechanisms, including VEGF and TNF-alpha secretion. The M2 macrophage phenotype, promoted by IL-4, IL-13, IL-10 and M-CSF, appears to contribute to immune suppression through the production of IL-10 and TGF-β [28]. Present findings are in line with the protumor function of M2 CD163^+^ macrophages that in combination CD8^+^ T cells represent predictive prognostic biosignatures in BRAF^V600^-mutated patients receiving MAPKi. However, they point on the key predictive role of M2 macrophage level outside and, more importantly, inside the tumor at baseline, before the treatment initiation.

This study presents some strengths: i) patients have been enrolled and treated homogeneously in IMI centers; ii) the majority of enrolled and investigated metastatic MPs were (122/158, 77%) in latest metastatic samples, thus reducing the potential discordance between primary and metastatic samples and to better reflect the actual immune biological status of the patient cohort; iii) semi-automated counting upon digital image acquisition, which allows unbiased and rapid quantification of the immune infiltrate in immunostained tissue sections and minimizes significant user errors due to categorical rankings was adopted; IV) since prospective clinical trials have demonstrated that single-agent BRAFi and BRAFi+MEKi have different response rates, PFS, and OS, we addressed this potential bias by accounting for the difference in treatments in the multivariate model, V) our findings were validated in an independent patient cohort, strictly following the Remark checklist [29]. However, we are aware of the study limitations, including: i) the retrospective nature of the analysis of prospective collected cohorts of patients, ii) overall, the time schedule for disease assessment was similar but not absolutely overlapping in all patients; iii) complex highly pigmented or necrotic metastatic melanoma tissues in which macrophages overlap or fuse together with pigmented melanoma cells forming densely packed layers of cells were seldom present. Although careful correlation with cell morphology and accurate identification of viable representative tumor areas were performed, this may represent a confounding factor that was addressed by optical microscopic evaluation. Another point is worthy to be underlined: in our cohort of metastatic lymph nodes, scoring evaluation did not differ from the other metastatic sites and positivity for the selected markers was evaluated within the tumor (intratumorally) as well as at the interface between the tumor and immune stroma (peritumorally). Nevertheless, the immunologic environment in the lymph node is peculiar and the crosstalk between specific subsets of lymphocytes and macrophages in different anatomical lymph node compartments may likely yield biological insights not globally applicable to other metastatic sites.

In our study, the main comparison was between the extreme categories high CD8^+^/low CD163^+^ and low CD8^+^/high CD163^+^, and the results of the categories in between (both low or both high) were instrumental only to confirm the trend of the risk in the three analyzed groups. The threshold for statistical significance was set at 0.05, and no adjustment for multiple tests was planned. The purpose of our study was to evaluate the impact of a limited number of biomarkers on prognosis, and these biomarkers should be prospectively validated in large clinical studies. Nevertheless, the robustness of our results was tested by including a validation cohort.

## Conclusions

Our findings indicate that a specific preexisting profile of T and macrophage distribution inside and outside melanoma dictates the level of resistance to MAPKi. Our results could have important implications for clinical therapeutic strategies. Since patients with absent/low intratumoral infiltration of CD8^+^ cells and high intratumoral CD163^+^ cells have a statistically significant lower ORR and shorter OS, they should deserve a different therapeutic strategy. Whether the hostile immune microenvironment induced by accumulated macrophages can be overcome by either inhibiting macrophage polarization to a M2 phenotype or targeting the inflammatory signaling promoted by NF-kB with IkB kinase inhibitors is currently unknown. Additional strategies can include the colony-stimulating factor (CSF)-1R inhibitor PLX3397 that has been shown to reduce myeloid cell infiltration and enhance adoptive cell transfer immunotherapy in BRAF^V600E^-driven melanoma genesis in mice [30]. Our findings along with other translational studies support the proposal to design new ad hoc prospective clinical trials in order to improve long-term survival of advanced MPs receiving MAPKi. In addition, the present study further underlines that a better understanding of the mechanisms that control the recruitment of immune cells in the TME and their distribution in the intra- and peritumoral space is essential to devise better therapeutic options in metastatic MPs, and particularly in those undergoing treatment with MAPKi.

## Supplementary information

**Additional file 1: Table S1.** Patients’ characteristics. **Table S2.** Associations. **Table S3.** Tissue biomarkers – combination. **Table S4.** Multivariable logistic model - Response rate. **Table S5.** Multivariable Cox regression model – Survival. **Table S6.** Validation cohort. **Table S7.** Validation cohort - Multivariable Cox regression model – Impact of tissue biomarkers on progression free survival and overall survival – validation cohort.

**Additional file 2: Figure S1.** Immunohistochemistry with anti-PD-L1 antibody shows positivity in more than 5% of tumor cells at membranous level (A). Immunohistochemistry with anti-PD-L2 antibody shows negative tumor cells with internal positive control (B). (original magnification 10x, scale bar 100 μm, inset 40x, scale bar 20 μm). **Figure S2.** Immunohistochemical β-catenin expression in metastatic melanoma tissues. At subcellular level, immunoreactivity is observed in the cytoplasm and scattered nuclei (A) and membrane and nuclear (B). (original magnification 10x, scale bar 100 μm, inset 40x, scale bar 20 μm). **Figure S3.** Distribution patterns and density of intratumoral and peritumoral CD8^+^ T and CD163^+^ cells in the training cohort. Low: score = 0, 1+; High: score = 2+,3 + .

## Data Availability

Not applicable.

## Abbreviations

AJCC: American Joint Committee on Cancer
BRAFi: BRAF inhibitors
CR: complete response
ECOG-PS: Eastern Cooperative Oncology Group performance status
FFPE: Formalin fixed paraffin-embedded
HR: hazard ratio
IMI: Italian Melanoma Intergroup
LDH: lactate dehydrogenase
MAPKi: MAPK inhibitors
MPs: melanoma patients
NE: not evaluable
OR: odd ratio
ORR: Overall response rate
OS: overall survival
PD: progressing disease
PFS: progression free survival
RGB: red, green, blue.
TME: tumor microenvironment
TNM: Tumor, Node, Metastasis
