# Supplementary material for: The density and spatial tissue distribution of CD8+ and CD163+ immune cells predict response and outcome in melanoma patients receiving MAPK inhibitors
Source: J Immunother Cancer. 2019 Nov 15;7:308. doi: 10.1186/s40425-019-0797-4 (PMC6858711; doi:10.1186/s40425-019-0797-4)
Supplement: Supplementary file 1 — Additional file 1: Table S1. Patients’ characteristics. Table S2. Associations. Table S3. Tissue biomarkers – combination. Table S4. Multivariable logistic model - Response rate. Table S5. Multivariable Cox regression model – Survival. Table S6. Validation cohort. Table S7. Validation cohort - Multivariable Cox regression model – Impact of tissue biomarkers on progression free survival and overall survival – validation cohort. [file 40425_2019_797_MOESM1_ESM.docx]

**The density and spatial tissue distribution of CD8+ and CD163+ immune cells predict response and outcome in melanoma patients receiving MAPKi**

Daniela Massi^1^, et al. on behalf of the Italian Melanoma Intergroup

**Additional file 1**

**Table S1.** Patients’ characteristics

**Table S2.** Associations

**Table S3.** Tissue biomarkers - combination

**Table S4.** Multivariable logistic model - Response rate

**Table S5.** Multivariable Cox regression model - Survival

**Table S6.** Validation cohort

**Table S7.** Validation cohort - Multivariable Cox regression model – Impact of tissue biomarkers on progression free survival and overall survival – validation cohort

**Table S1. Patients’ characteristics**

|  | **OVERALL N=158** |
| --- | --- |
| **Male Sex** | 95 (60.1) |
| **Age** Median (Q1 - Q3) | 59.2 (47.7-70.7) |
| **Stage** |  |
| M1a | 42 (26.6) |
| M1b | 21 (13.3) |
| M1c | 95 (60.1) |
| **LDH** |  |
| High (>UNL) | 44 (32.8) |
| Normal | 90 (67.2) |
| Missing | 24 |
| **ECOG PS** |  |
| 0 | 112 (71.3) |
| 1 | 36 (22.9) |
| 2 | 7 (4.5) |
| 3 | 2 (1.3) |
| Missing | 1 |

**Legend:** Q1: first quartile, Q3: third quartile, PS: Performance Status

**Table S2. Associations**

|  | **PD-L1** | **iCD8 ^+^** | **pCD8^+^** | **iCD163^+^** | **pCD163^+^** | **β-catenin M** | **β-catenin C** | **β-catenin N** | **Stage** | **LDH** | **Sex** | **PS** |
| --- | --- | --- | --- | --- | --- | --- | --- | --- | --- | --- | --- | --- |
| **iCD8^+^** |  |  | **<.0001** | 0.855 | 0.964 |  |  |  | 0.873 | 0.684 | 0.638 | 0.798 |
| **pCD8^+^** |  |  |  | 0.390 | 0.097 |  |  |  |  |  |  |  |
| **iCD163^+^** |  |  |  |  | **<.0001** |  |  |  | 0.998 | 0.233 | 0.318 | 0.221 |
| **PD-L1** |  | 0.158 | 0.210 | **0.008** | **0.032** |  |  |  | 0.298 | 0.718 | 0.579 | 0.259 |
| **PD-L2** | **0.018** | 0.458 | 0.636 | 0.878 | 0.837 | 0.149 | 0.484 | 0.135 | 0.518 | 0.109 | 0.972 | 0.689 |
| **β-catenin M** | 0.358 | 0.831 | 0.282 | 0.197 | 0.293 |  | **<.0001** | 0.074 | 0.597 | 0.141 | 0.581 | 0.101 |
| **β-catenin C** | 0.379 | 0.099 | 0.805 | 0.924 | 0.163 |  |  | **0.008** | 0.924 | 0.707 | 0.446 | 0.144 |
| **β-catenin N** | 0.266 | 1.000 | 0.516 | 0.246 | 0.915 |  |  |  | 0.939 | 0.821 | 0.174 | 0.047 |

*p-values for chi-square test*

**Legend:** i: intratumoral; p: peritumoral; M: membranous; N: nuclear, C: cytoplasmic, PS: Performance Status

**Table S3. Tissue biomarkers - combination**

|  | **OVERALL N=158** |
| --- | --- |
| **iCD8^+^/iCD163^+^** |  |
| iCD8^+^ high - iCD163^+^ low | 25 (18.5) |
| iCD8^+^ high - iCD163^+^ high/ iCD8^+^ low - iCD163^+^ low | 68 (50.4) |
| iCD8^+^ low - iCD163^+^ high | 42 (31.1) |
| Missing | 23 |
| **pCD8^+^/pCD163^+^** |  |
| pCD8^+^ high - pCD163^+^ low | 46 (34.8) |
| pCD8^+^ high - pCD163^+^ high/ pCD8^+^ low - pCD163^+^ low | 61 (46.2) |
| pCD8^+^ low - pCD163^+^ high | 25 (18.9) |
| Missing | 26 |
| **PD-L1/iCD8^+^** |  |
| PD-L1 **≥**5% - iCD8^+^ low | 33 (25.4) |
| PD-L1 **≥**5% - iCD8^+^ high /PD-L1<5% - iCD8^+^ low | 74 (56.9) |
| PD-L1 <5% - iCD8^+^ high | 23 (17.7) |
| Missing | 28 |
| **PD-L1/pCD8^+^** |  |
| PD-L1 **≥**5% - pCD8^+^ low | 15 (11.6) |
| PD-L1 **≥**5% - pCD8^+^ high /PD-L1<5% - pCD8^+^ low | 69 (53.5) |
| PD-L1 <5% - pCD8^+^ high | 45 (34.9) |
| Missing | 29 |
| **β-catenin M/iCD8^+^** |  |
| iCD8^+^ low - β-catenin overexpressed | 36 (27.3) |
| iCD8^+^ high - β-catenin overexpressed / iCD8^+^ low - β-catenin negative | 67 (50.8) |
| iCD8^+^ high - β-catenin negative | 29 (22.0) |
| Missing | 26 |
| **β-catenin M/pCD8^+^** |  |
| pCD8^+^ low - β-catenin overexpressed | 15 (11.5) |
| pCD8^+^ high - β-catenin overexpressed / pCD8^+^ low - β-catenin negative | 68 (51.9) |
| pCD8^+^ high - β-catenin negative | 48 (36.6) |
| Missing | 27 |
| **β-catenin N/iCD8^+^** |  |
| iCD8^+^ low - β-catenin overexpressed | 8 (6.2) |
| iCD8^+^ high - β-catenin overexpressed / iCD8^+^ low - β-catenin negative | 77 (59.2) |
| iCD8^+^ high - β-catenin negative | 45 (34.6) |
| Missing | 28 |
| **β-catenin N/pCD8^+^** |  |
| pCD8^+^ low - β-catenin overexpressed | 3 (2.3) |
| pCD8^+^ high - β-catenin overexpressed / pCD8^+^ low - β-catenin negative | 47 (36.4) |
| pCD8^+^ high - β-catenin negative | 79 (61.2) |
| Missing | 29 |
| **β-catenin M/iCD8^+^** |  |
| iCD8^+^ low - β-catenin overexpressed | 45 (34.1) |
| iCD8^+^ high - β-catenin overexpressed / iCD8^+^ low - β-catenin negative | 57 (43.2) |
| iCD8^+^ high - β-catenin negative | 30 (22.7) |
| Missing | 26 |
| **β-catenin C/pCD8^+^** |  |
| pCD8^+^ low - β-catenin overexpressed | 21 (16.0) |
| pCD8^+^ high - β-catenin overexpressed / pCD8^+^ low - β-catenin negative | 64 (48.9) |
| pCD8^+^ high - β-catenin negative | 46 (35.1) |
| Missing | 27 |
| **Legend:**i: intratumoral; p: peritumoral; M: membranous; N: nuclear; C: cytoplasmic - CD8^+^/CD163^+^ low: score 0-1+; high: score 2+3+; β-catenin M negative: lower than 60%; β-catenin M overexpressed: equal or higher than 60%; β-catenin N negative: lower than 10%; β-catenin N overexpressed: equal or higher than 10%; β-catenin C negative: equal to 0; β-catenin C overexpressed: higher than 0 | |

**Table S4. Multivariable logistic model – Impact of all tissue biomarkers on response rate**

|  | **N** | **P-value of variable** | **OR (95% CI)** | **P-value of contrasts** |
| --- | --- | --- | --- | --- |
| **iCD8^+^ (high vs low)** | 121 | 0.074 | 2.15 ( 0.93 - 4.98 ) |  |
| **pCD8^+^ (high vs low)** | 120 | 0.477 | 0.73 ( 0.31 - 1.73 ) |  |
| **iCD163^+^ (high vs low)** | 128 | 0.003* | 0.28 ( 0.12 - 0.65 ) |  |
| **pCD163^+^ (high vs low)** | 124 | 0.136 | 0.50 ( 0.20 - 1.24 ) |  |
| **PD-L1 continuous** | 125 | 0.622 | 0.99 ( 0.98 - 1.02 ) |  |
| **PD-L1 (≥5% vs <5%)** | 126 | 0.455 | 0.75 ( 0.35 - 1.61 ) |  |
| **PD-L2 continuous** | 122 | 0.787 | 1.00 ( 0.96 - 1.03 ) |  |
| **PD-L2 (≥5% vs <5%)** | 123 | 0.839 | 1.14 ( 0.32 - 4.08 ) |  |
| **β-catenin M continuous** | 129 | 0.086 | 0.99 ( 0.98 - 1.00 ) |  |
| **β-catenin M (overexpressed vs negative)** | 129 | 0.068 | 0.48 ( 0.21 - 1.06 ) |  |
| **β-catenin C continuous** | 129 | 0.396 | 1.00 ( 0.99 - 1.01 ) |  |
| **β-catenin C (overexpressed vs negative)** | 129 | 0.214 | 0.61 ( 0.27 - 1.34 ) |  |
| **β-catenin N continuous** | 127 | 0.067 | 0.97 ( 0.95 - 1.00 ) |  |
| **β-catenin N (overexpressed vs negative)** | 127 | 0.094 | 0.34 ( 0.10 - 1.20 ) |  |
| **PD-L1 - iCD8^+^** | 115 | 0.312 |  |  |
| PD-L1 **≥**5% - iCD8^+^ low |  |  | reference |  |
| PD-L1 **≥**5% - iCD8^+^ high /PD-L1<5% - iCD8^+^ low |  |  | 1.77 ( 0.68 - 4.58 ) | 0.242 |
| PD-L1 <5% - iCD8^+^ high |  |  | 2.58 ( 0.68 - 9.77 ) | 0.163 |
| **PD-L1 - pCD8^+^** | 114 | 0.355 |  |  |
| PD-L1 **≥**5% - pCD8^+^ low |  |  | reference |  |
| PD-L1 **≥**5% - pCD8^+^ high /PD-L1<5% - pCD8^+^ low |  |  | 2.01 ( 0.58 - 6.94 ) | 0.272 |
| PD-L1 <5% - pCD8^+^ high |  |  | 1.15 ( 0.31 - 4.22 ) | 0.833 |
| **iCD8^+^ - iCD163^+^** | 117 | 0.004* |  |  |
| iCD8^+^ low - iCD163^+^ high |  |  | reference |  |
| iCD8^+^ high - iCD163^+^ high/ iCD8^+^ low -iCD163^+^ low |  |  | 3.39 ( 1.29 - 8.96 ) | 0.014* |
| iCD8^+^ high - iCD163^+^ low |  |  | 9.91 ( 2.23 - 44.0 ) | 0.003* |
| **pCD8^+^ - pCD163^+^** | 113 | 0.136 |  |  |
| pCD8^+^ low - pCD163^+^ high |  |  | reference |  |
| pCD8^+^ high - pCD163^+^ high/ pCD8^+^ low -pCD163^+^ low |  |  | 1.78 ( 0.58 - 5.50 ) | 0.315 |
| pCD8^+^ high - pCD163^+^ low |  |  | 3.36 ( 1.01 - 11.1 ) | 0.048* |
| **β-catenin M - iCD8^+^** | 117 | 0.016* |  |  |
| iCD8^+^ low - β -catenin overexpressed |  |  | reference |  |
| iCD8^+^ high - β-catenin overexpressed / iCD8^+^ low - β-catenin negative |  |  | 2.39 ( 0.92 - 6.24 ) | 0.074 |
| iCD8^+^ high - β-catenin negative |  |  | 7.45 ( 1.85 - 30.0 ) | 0.005* |
| **β-catenin M - pCD8^+^** | 116 | 0.408 |  |  |
| pCD8^+^ low - β-catenin overexpressed |  |  | reference |  |
| pCD8^+^ high - β-catenin overexpressed / pCD8^+^ low - β-catenin negative |  |  | 1.20 ( 0.34 - 4.21 ) | 0.772 |
| pCD8^+^ high - β-catenin negative |  |  | 2.11 ( 0.54 - 8.19 ) | 0.282 |
| **β-catenin C - iCD8^+^** | 117 | 0.172 |  |  |
| iCD8^+^ low - β-catenin overexpressed |  |  | reference |  |
| iCD8^+^ high - β-catenin overexpressed / iCD8^+^ low - β-catenin negative |  |  | 2.23 ( 0.87 - 5.68 ) | 0.093 |
| iCD8^+^ high - β-catenin negative |  |  | 2.38 ( 0.78 - 7.28 ) | 0.129 |
| **β-catenin C - pCD8^+^** | 116 | 0.410 |  |  |
| pCD8^+^ low - β-catenin overexpressed |  |  | reference |  |
| pCD8^+^ high - β-catenin overexpressed / pCD8^+^ low - β-catenin negative |  |  | 1.98 ( 0.64 - 6.16 ) | 0.235 |
| pCD8^+^ high - β-catenin negative |  |  | 1.22 ( 0.38 - 3.97 ) | 0.736 |
| **β-catenin N - iCD8^+^** | 115 | 0.065 |  |  |
| iCD8^+^ low - β-catenin overexpressed |  |  | reference |  |
| iCD8^+^ high - β-catenin overexpressed / iCD8^+^ low - β-catenin negative |  |  | 5.63 ( 0.91 - 34.6 ) | 0.062 |
| iCD8^+^ high - β-catenin negative |  |  | 9.54 ( 1.41 - 64.6 ) | 0.021* |
| **β-catenin N - pCD8^+^** | 114 | 0.707 |  |  |
| pCD8^+^ low - β-catenin overexpressed |  |  | reference |  |
| pCD8^+^ high - β-catenin overexpressed / pCD8^+^ low - β-catenin negative |  |  | 2.95 ( 0.21 - 40.5 ) | 0.419 |
| pCD8^+^ high - β-catenin negative |  |  | 2.52 ( 0.19 - 33.7 ) | 0.484 |
| **Legend:**All the analyses were adjusted for Stage, LDH, PS, treatment (BRAFi+MEKi vs BRAFi); i: intratumoral; p: peritumoral**;** M: membranous; N: nuclear, C: cytoplasmic; CD8^+^/CD163^+^ low: score 0,1+; high: score 2+3+; β-catenin M negative: lower than 60%; β-catenin M overexpressed: equal or higher than 60%; β-catenin N negative: lower than 10%; β-catenin N overexpressed: equal or higher than 10%; β-catenin C negative: equal to 0; β-catenin C overexpressed: higher than 0 | | | | |

**Table S5. Multivariable Cox regression model – Impact of all biomarkers on progression free and overall survival**

|  |  | **Progression free survival*** | | | **Overall survival**** | | |
| --- | --- | --- | --- | --- | --- | --- | --- |
|  | **N** | **P-value of variable** | **HR (95% CI)** | **P-value of contrasts** | **P-value of variable** | **HR (95% CI)** | **P-value of contrasts** |
| **iCD8^+^ (high vs low)** | 122 | 0.346 | 0.82 ( 0.54 - 1.24 ) |  | 0.072 | 0.65 ( 0.41 - 1.04 ) |  |
| **pCD8^+^ (high vs low)** | 121 | 0.901 | 1.03 ( 0.66 - 1.60 ) |  | 0.778 | 0.93 ( 0.58 - 1.50 ) |  |
| **iCD163^+^ (high vs low)** | 129 | 0.283 | 1.25 ( 0.83 - 1.88 ) |  | 0.129 | 1.42 ( 0.90 - 2.22 ) |  |
| **pCD163^+^ (high vs low)** | 125 | 0.201 | 1.34 ( 0.86 - 2.11 ) |  | 0.126 | 1.49 ( 0.89 - 2.48 ) |  |
| **PD-L1 continuous** | 126 | 0.297 | 0.99 ( 0.98 - 1.01 ) |  | 0.327 | 0.99 ( 0.98 - 1.01 ) |  |
| **PD-L1 (≥5% vs <5%)** | 127 | 0.571 | 0.89 ( 0.59 - 1.34 ) |  | 0.788 | 0.94 ( 0.60 - 1.48 ) |  |
| **PD-L2 continuous** | 123 | 0.690 | 1.00 ( 0.97 - 1.02 ) |  | 0.676 | 1.00 ( 0.98 - 1.02 ) |  |
| **PD-L2 (≥5% vs <5%)** | 124 | 0.265 | 0.68 ( 0.34 - 1.35 ) |  | 0.180 | 0.61 ( 0.29 - 1.26 ) |  |
| **β-catenin M continuous** | 130 | 0.107 | 1.00 ( 1.00 - 1.01 ) |  | 0.323 | 1.00 ( 1.00 - 1.01 ) |  |
| **β-catenin M (overexpressed vs negative)** | 130 | 0.362 | 1.21 ( 0.80 - 1.83 ) |  | 0.849 | 1.04 ( 0.67 - 1.62 ) |  |
| **β-catenin C continuous** | 130 | 0.170 | 1.00 ( 1.00 - 1.01 ) |  | 0.109 | 1.00 ( 1.00 - 1.01 ) |  |
| **β -catenin C (overexpressed vs negative)** | 130 | 0.051 | 1.50 ( 1.00 - 2.26 ) |  | 0.059 | 1.52 ( 0.98 - 2.36 ) |  |
| **β-catenin N continuous** | 128 | 0.281 | 1.01 ( 0.99 - 1.02 ) |  | 0.068 | 1.01 ( 1.00 - 1.02 ) |  |
| **β-catenin N (overexpressed vs negative)** | 128 | 0.608 | 1.18 ( 0.63 - 2.22 ) |  | 0.918 | 1.04 ( 0.52 - 2.07 ) |  |
| **PD-L1 - iCD8^+^** | 116 | 0.873 |  |  | 0.362 |  |  |
| PD-L1 **≥**5% - iCD8^+^ low |  |  | reference |  |  | reference |  |
| PD-L1 **≥**5% - iCD8^+^ high /PD-L1<5% - iCD8^+^ low |  |  | 0.87 ( 0.53 - 1.45 ) | 0.603 |  | 0.90 ( 0.54 - 1.52 ) | 0.705 |
| PD-L1 <5% - iCD8^+^ high |  |  | 0.92 ( 0.49 - 1.74 ) | 0.805 |  | 0.57 ( 0.26 - 1.24 ) | 0.157 |
| **PD-L1 - pCD8^+^** | 115 | 0.322 |  |  | 0.833 |  |  |
| PD-L1 **≥**5% - pCD8^+^ low |  |  | reference |  |  | reference |  |
| PD-L1 **≥**5% - pCD8^+^ high /PD-L1<5% - pCD8^+^ low |  |  | 0.82 ( 0.44 - 1.54 ) | 0.540 |  | 0.92 ( 0.47 - 1.81 ) | 0.816 |
| PD-L1 <5% - pCD8^+^ high |  |  | 1.17 ( 0.60 - 2.27 ) | 0.651 |  | 1.08 ( 0.51 - 2.27 ) | 0.836 |
| **iCD8^+^ - iCD163^+^** | 118 | 0.010* |  |  | 0.002* |  |  |
| iCD8^+^ low - iCD163^+^ high |  |  | reference |  |  | reference |  |
| iCD8^+^ high - iCD163^+^ high/ iCD8^+^ low -iCD163^+^ low |  |  | 0.48 ( 0.30 - 0.78 ) | 0.003* |  | 0.46 ( 0.27 - 0.76 ) | 0.003* |
| iCD8^+^ high - iCD163^+^ low |  |  | 0.55 ( 0.30 - 1.03 ) | 0.063 |  | 0.34 ( 0.16 - 0.72 ) | 0.005* |
| **pCD8^+^ - pCD163^+^** | 114 | 0.117 |  |  | 0.021* |  |  |
| pCD8^+^ low - pCD163^+^ high |  |  | reference |  |  | reference |  |
| pCD8^+^ high - pCD163^+^ high/ pCD8^+^ low -pCD163^+^ low |  |  | 0.55 ( 0.31 - 0.97 ) | 0.038* |  | 0.43 ( 0.23 - 0.79 ) | 0.006* |
| pCD8^+^ high - pCD163^+^ low |  |  | 0.67 ( 0.37 - 1.23 ) | 0.196 |  | 0.52 ( 0.27 - 1.00 ) | 0.052 |
| **β-catenin M - iCD8^+^** | 118 | 0.279 |  |  | 0.310 |  |  |
| iCD8^+^ low - β-catenin overexpressed |  |  | reference |  |  | reference |  |
| iCD8^+^ high - β-catenin overexpressed / iCD8^+^ low - β-catenin negative |  |  | 0.69 ( 0.42 - 1.14 ) | 0.146 |  | 0.70 ( 0.41 - 1.19 ) | 0.183 |
| iCD8^+^ high - β-catenin negative |  |  | 0.65 ( 0.35 - 1.21 ) | 0.173 |  | 0.63 ( 0.33 - 1.23 ) | 0.176 |
| **β-catenin M - pCD8^+^** | 117 | 0.462 |  |  | 0.092 |  |  |
| pCD8^+^ low - β-catenin overexpressed |  |  | reference |  |  | reference |  |
| pCD8^+^ high - β-catenin overexpressed / pCD8^+^ low - β-catenin negative |  |  | 0.66 ( 0.33 - 1.28 ) | 0.217 |  | 0.45 ( 0.22 - 0.94 ) | 0.033* |
| pCD8^+^ high - β-catenin negative |  |  | 0.68 ( 0.34 - 1.40 ) | 0.299 |  | 0.61 ( 0.29 - 1.29 ) | 0.197 |
| **β-catenin C - iCD8^+^** | 118 | 0.212 |  |  | 0.098 |  |  |
| iCD8^+^ low - β-catenin overexpressed |  |  | reference |  |  | reference |  |
| iCD8^+^ high - β-catenin overexpressed / iCD8^+^ low - β-catenin negative |  |  | 0.70 ( 0.42 - 1.15 ) | 0.156 |  | 0.63 ( 0.37 - 1.06 ) | 0.084 |
| iCD8^+^ high - β-catenin negative |  |  | 0.63 ( 0.35 - 1.11 ) | 0.108 |  | 0.55 ( 0.30 - 1.00 ) | 0.052 |
| **β-catenin C - pCD8^+^** | 117 | 0.416 |  |  | 0.046* |  |  |
| pCD8^+^ low - β-catenin overexpressed |  |  | reference |  |  | reference |  |
| pCD8^+^ high - β-catenin overexpressed / pCD8^+^ low - β-catenin negative |  |  | 0.69 ( 0.37 - 1.29 ) | 0.248 |  | 0.44 ( 0.23 - 0.85 ) | 0.014* |
| pCD8^+^ high - β-catenin negative |  |  | 0.65 ( 0.33 - 1.26 ) | 0.201 |  | 0.53 ( 0.27 - 1.02 ) | 0.059 |
| **β-catenin N - iCD8^+^** | 116 | 0.306 |  |  | 0.267 |  |  |
| iCD8^+^ low - β-catenin overexpressed |  |  | reference |  |  | reference |  |
| iCD8^+^ high - β-catenin overexpressed / iCD8^+^ low - β-catenin negative |  |  | 0.63 ( 0.28 - 1.41 ) | 0.259 |  | 0.70 ( 0.29 - 1.72 ) | 0.441 |
| iCD8^+^ high - β-catenin negative |  |  | 0.52 ( 0.22 - 1.22 ) | 0.132 |  | 0.51 ( 0.20 - 1.32 ) | 0.163 |
| **β-catenin N - pCD8^+^** | 115 | 0.890 |  |  | 0.715 |  |  |
| pCD8^+^ low - β-catenin overexpressed |  |  | reference |  |  | reference |  |
| pCD8^+^ high - β-catenin overexpressed / pCD8^+^ low - β-catenin negative |  |  | 0.90 ( 0.26 - 3.04 ) | 0.861 |  | 0.61 ( 0.18 - 2.12 ) | 0.438 |
| pCD8^+^ high - β-catenin negative |  |  | 0.82 ( 0.24 - 2.77 ) | 0.745 |  | 0.60 ( 0.17 - 2.06 ) | 0.414 |
| **Legend: ***Adjusted for Stage, LDH, PS, treatment (BRAFi+MEKi vs BRAFi); ** Adjusted for Stage, LDH, PS, treatment (BRAFi+MEKi vs BRAFi), Immunotherapy; i: intratumoral; p: intratumoral;  M: membranous; N: nuclear; C: cytoplasmic; CD8^+^/ CD163^+^ low: score 0,1+; high: score 2+3+; β-catenin M negative: lower than 60%; β-catenin M overexpressed: equal or higher than 60%; β-catenin N negative: lower than 10%; β-catenin N overexpressed: equal or higher than 10%; β-catenin C negative: equal to 0; β-catenin C overexpressed: higher than 0 | | | | | | | |

**Table S6. Validation cohort**

|  | **OVERALL**  **N=55** |
| --- | --- |
| **Sex** |  |
| Female | 25 (45.5) |
| Male | 30 (54.5) |
| **Age at diagnosis of metastatic disease** |  |
| Median (Q1- Q3) | 53 (43-63) |
| **Stage** |  |
| IV M1a | 12 (21.8) |
| IV M1b | 13 (23.6) |
| IV M1c | 30 (54.5) |
| **Treatment** |  |
| BRAF | 35 (63.6) |
| BRAF+MEK | 20 (36.4) |
| **Subsequent immunotherapy** |  |
| No | 43 (78.2) |
| Yes | 12 (21.8) |
| **CD8 peri** |  |
| Low (0, 1+) | 31 (56.4) |
| High (2+,3+) | 24 (43.6) |
| **CD8 intra** |  |
| Low (0, 1+) | 33 (60.0) |
| High (2+,3+) | 22 (40.0) |
| **CD163 peri** |  |
| Low (0, 1+) | 35 (63.6) |
| High (2+,3+) | 20 (36.4) |
| **CD163 intra** |  |
| Low (0, 1+) | 24 (43.6) |
| High (2+,3+) | 31 (56.4) |
| **CD8 /CD163 peri** |  |
| CD8 2+,3+, CD163 0, 1+ | 18 (32.7) |
| CD8 2+,3+, CD163 2+, 3+/ CD8 0, 1+, CD163 0, 1+ | 23 (41.8) |
| CD8 0, 1+, CD163 2+, 3+ | 14 (25.5) |
| **CD8 /CD163 intra** |  |
| CD8 2+,3+, CD163 0, 1+ | 15 (27.3) |
| CD8 2+,3+, CD163 2+, 3+/ CD8 0, 1+, CD163 0, 1+ | 16 (29.1) |
| CD8 0, 1+, CD163 2+, 3+ | 24 (43.6) |
| **Legend:**Q1: first quartile, Q3: third quartile | |

**Table S7. Validation cohort - Multivariable Cox regression model – Impact of tissue biomarkers on progression free survival and overall survival – validation cohort**

|  |  | **Progression free survival^#^** | | | **Overall survival^##^** | | |
| --- | --- | --- | --- | --- | --- | --- | --- |
|  | **N** | **P-value of variable** | **HR (95% CI)** | **P-value of contrasts** | **P-value of variable** | **HR (95% CI)** | **P-value of contrasts** |
| **iCD8^+^ (high vs low)** | 55 | <0.001* | 0.12 ( 0.05 - 0.27 ) |  | 0.016* | 0.14 ( 0.03 - 0.69 ) |  |
| **pCD8^+^ (high vs low)** | 55 | <0.001* | 0.17 ( 0.08 - 0.38 ) |  | 0.064 | 0.26 ( 0.06 - 1.08 ) |  |
| **iCD163^+^ (high vs low)** | 55 | 0.002* | 3.07 ( 1.53 - 6.17 ) |  | 0.046* | 4.31 ( 1.03 - 18.1 ) |  |
| **pCD163^+^ (high vs low)** | 55 | 0.001* | 3.00 ( 1.54 - 5.84 ) |  | 0.033* | 4.28 ( 1.13 - 16.2 ) |  |
| **iCD8^+^ - iCD163^+^** | 55 | <0.001* |  |  | 0.041* |  |  |
| iCD8^+^ low - iCD163^+^ high |  |  | reference |  |  | reference |  |
| iCD8^+^ high - iCD163^+^ high/ iCD8^+^ low - iCD163^+^ low |  |  | 0.16 ( 0.06 - 0.42 ) | <0.001* |  | 0.15 ( 0.02 - 1.28 ) | 0.082 |
| iCD8^+^ high - iCD163^+^ low |  |  | 0.05 ( 0.01 - 0.15 ) | <0.001* |  | 0.04 ( 0.00 - 0.50 ) | 0.013* |
| **pCD8^+^ - pCD163^+^** | 55 | <0.001* |  |  | 0.062 |  |  |
| pCD8^+^ low - pCD163^+^ high |  |  | reference |  |  | reference |  |
| pCD8^+^ high - pCD163^+^ high/ pCD8^+^ low -pCD163^+^ low |  |  | 0.64 ( 0.31 - 1.34 ) | 0.241 |  | 0.36 ( 0.09 - 1.51 ) | 0.162 |
| pCD8^+^ high - pCD163^+^ low |  |  | 0.06 ( 0.02 - 0.21 ) | <0.001* |  | 0.12 ( 0.02 - 0.73 ) | 0.021* |
| **Legend:**^#^Adjusted for Stage, treatment (BRAFi+MEKi vs BRAFi); ^##^ Adjusted for Stage, treatment (BRAFi+MEKi vs BRAFi), subsequent Immunotherapy (yes/no); I: intratumoral; p: peritumoral; CD8^+^/ CD163^+^ low: score 0,1+, high: score 2+,3+. | | | | | | | |
