# Supplementary figures and images for: The density and spatial tissue distribution of CD8+ and CD163+ immune cells predict response and outcome in melanoma patients receiving MAPK inhibitors
Source: J Immunother Cancer. 2019 Nov 15;7:308. doi: 10.1186/s40425-019-0797-4 (PMC6858711; doi:10.1186/s40425-019-0797-4)

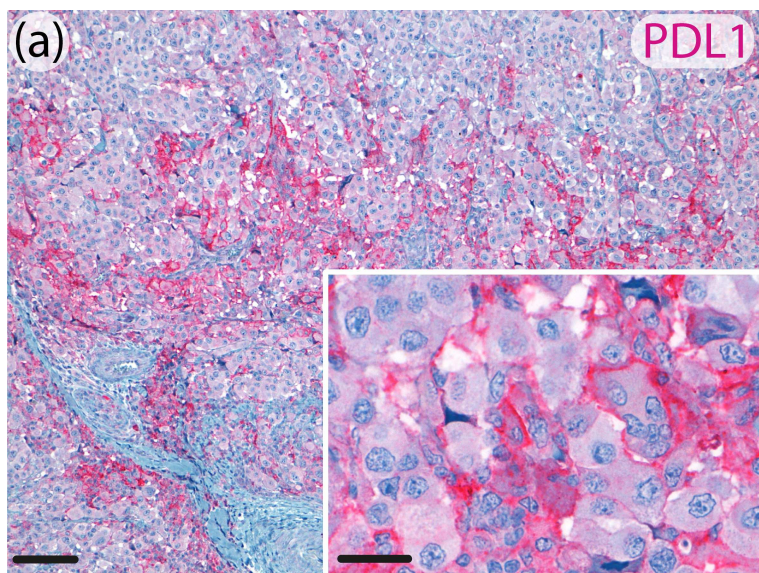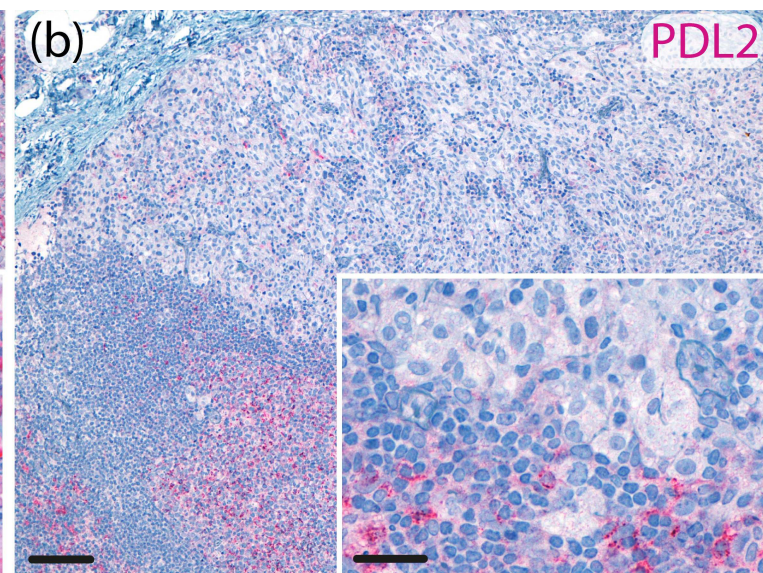

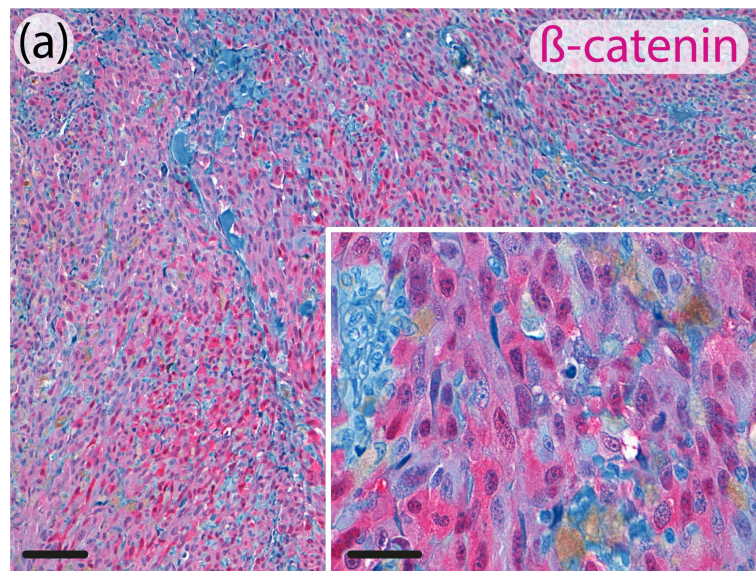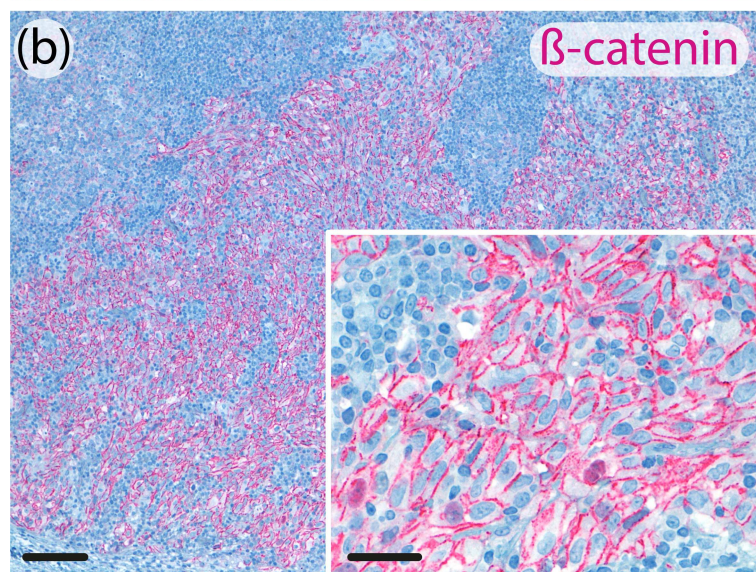

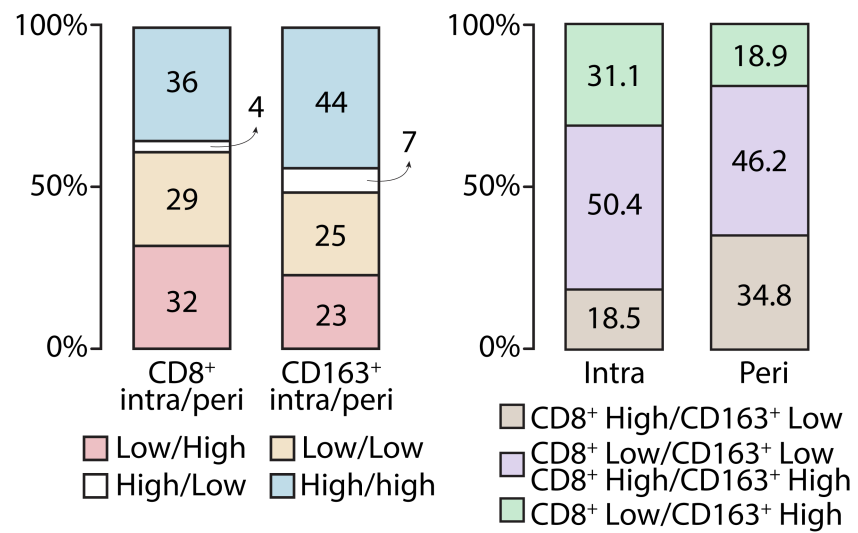

Supplement: Supplementary file 2 — Additional file 2: Figure S1. Immunohistochemistry with anti-PD-L1 antibody shows positivity in more than 5% of tumor cells at membranous level (A). Immunohistochemistry with anti-PD-L2 antibody shows negative tumor cells with internal positive control (B). (original magnification 10x, scale bar 100 μm, inset 40x, scale bar 20 μm). Figure S2. Immunohistochemical β-catenin expression in metastatic melanoma tissues. At subcellular level, immunoreactivity is observed in the cytoplasm and scattered nuclei (A) and membrane and nuclear (B). (original magnification 10x, scale bar 100 μm, inset 40x, scale bar 20 μm). Figure S3. Distribution patterns and density of intratumoral and peritumoral CD8+ T and CD163+ cells in the training cohort. Low: score = 0, 1+; High: score = 2+,3 + . [file 40425_2019_797_MOESM2_ESM.pdf]
